# Cobalt(II) and Manganese(II) Complexes of Sodium Monensinate A Bearing Nitrate Co-Ligands

**DOI:** 10.3390/ijms252212129

**Published:** 2024-11-12

**Authors:** Nikolay Petkov, Miroslav Boyadzhiev, Nikita Bozhilova, Petar Dorkov, Elzhana Encheva, Angel Ugrinov, Ivayla N. Pantcheva

**Affiliations:** 1 Faculty of Chemistry and Pharmacy, Sofia University “St. Kliment Ohridski”, 1164 Sofia, Bulgaria; ahnp@chem.uni-sofia.bg (N.P.); mirobo100@gmail.com (M.B.); eencheva@ipc.bas.bg (E.E.); 2 Faculty of Biology, Sofia University “St. Kliment Ohridski”, 1164 Sofia, Bulgaria; nikitobojilova@gmail.com; 3 Research and Development Department, Biovet Ltd., 4550 Peshtera, Bulgaria; p_dorkov@biovet.com; 4 Institute of Physical Chemistry, Bulgarian Academy of Sciences, 1113 Sofia, Bulgaria; 5 Department of Chemistry and Biochemistry, North Dakota State University, Fargo, ND 58105, USA

**Keywords:** polyether ionophore, heteronuclear mixed-ligand complex, crystal structure, antimicrobial activity

## Abstract

Monensic acid is a natural polyether ionophore and is a therapeutic of first choice in veterinary medicine for the control of coccidiosis. Although known as a sodium-binding ligand, it can also form a variety of coordination species depending on experimental conditions applied. In this study, we present the crystal structures and properties of Co(II) and Mn(II) complexes of sodium monensinate (MonNa) derived from the reaction of MonNa with cobalt or manganese dinitrates. The newly obtained coordination compounds have the same composition [M(MonNa)_2_(NO_3_)_2_] but the transition metal ions are placed in a different environment. The two nitrate ligands behave mono- or bidentately bound in the Co(II)- and Mn(II)-containing species, respectively, while the monensinate ligands act in a similar manner through their monodentate carboxylate functions. The formed CoO_4_ and MnO_6_ units determine the geometry of the corresponding inner coordination cores of the complexes as a tetrahedron in the case of Co(II), and as a strongly distorted octahedral structure in Mn(II) species. The effect of inorganic anions on the antibacterial performance of sodium monensinate appears to be negligible, while the presence of Co(II) or Mn(II) cations preserves or enhances the activity of unmodified MonNa, which differentially affects the growth of *Bacillus subtilis*, *Bacillus cereus*, *Kocuria rhizophila*, *Staphilococcus aureus*, and *Staphilococcus saprophyticus* strains.

## 1. Introduction

**Figure 1:**
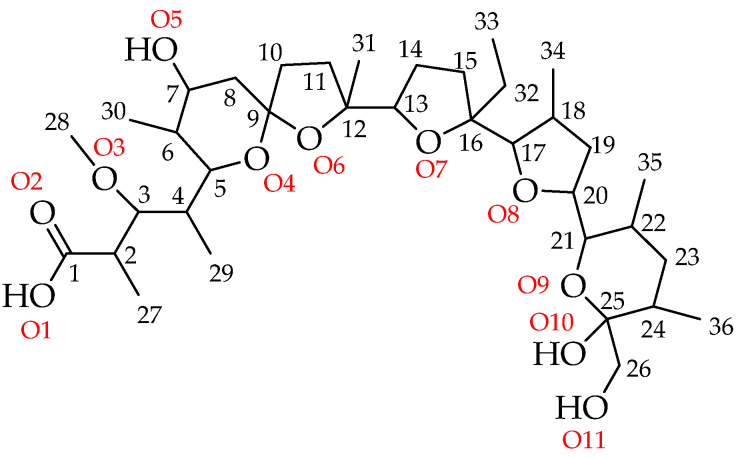
Chemical structure of monensic acid (MonH).

Monensic acid A (MonH, Figure 1) is an antiprotozoal agent isolated as a metabolic product of the cell-life cycle of *Streptomyces cinnamonensis*. First discovered in 1967 by Agtarap et al. [1], it pioneered the authentication of polyether ionophores, a group of compounds used in animal husbandry as coccidiostats and antibacterials [2,3,4,5,6,7,8,9,10,11,12]. In the last six to seven decades, more animals have been medicated with ionophores for the control of the disease than any other therapeutics in the history of veterinary medicine [13]. Written by Chapman et al. in 2010 on the occasion of “40 years of monensin”, this statement is still valid in the present day. Recently, some representatives of this chemical class are receiving special attention due to their pronounced antitumor properties, and “old” drugs are being repurposed for potentially new applications [14,15,16,17,18].

One of the strategies to modulate the activity of natural compounds is their modification by introducing appropriate functionalities that can increase the parent potential. Another option is based on coordination chemistry tools, where the incorporation of metal ions into the structure of ligands may positively change their biological properties. Both approaches have proven reliable in the search for more potent agents in numerous fields of medicinal chemistry [19,20,21,22,23,24,25,26].

Over the years, we have been interested in the modification of monensin and salinomycin by complexation with metal ions, having isolated a diversity of structures depending on the antibiotic form (acid or sodium derivative) and metal salt. The accumulated data reveal that the deprotonated ionophores ligate mostly in a bidentate coordination mode [27,28,29,30,31,32,33,34], while their metal(I) counterparts (particularly sodium monensinate, MonNa) construct rarely observed heteronuclear mixed-ligand coordination species with monodentately bound antibiotic [35,36,37]. The only few known examples include [M(MonNa)_2_Cl_2_] (M = Co, Mn, Cu) [35,36] and [Hg(MonNa)_2_(SCN)_2_] [37], whose solid-state structures have been explicitly analyzed by single-crystal X-ray diffraction (SCXRD). The formation of these complexes raised the question of whether sodium monensinate is able to react with metal(II) salts bearing different anions and how they can (possibly) affect the coordination environment of the central metal ion. Another point to consider is the influence of the counterion which may alter the biological properties of the coordination compounds.

As part of our studies to address the above-mentioned queries, we explored the behavior of MonNa in multiple systems comprising cobalt(II) and manganese(II) nitrates, perchlorates, and acetates. The results show that, among the selected anions, only nitrates successfully form new complex species and their properties are discussed in the present paper. The coordination compounds are of a general composition [M(MonNa)_2_(NO_3_)_2_], and, despite the similarities to the already reported [M(MonNa)_2_Cl_2_] [35], we will show that the substitution of chlorides with bulky nitrates leads to the nonequivalence of some donor atoms bound to the metal(II) centers. In addition to the structural and spectral characterization, the antimicrobial activity of newly obtained complexes and the parent antibiotic is evaluated against a set of five Gram-positive aerobic microorganisms.

## 2. Results and Discussion

The interaction of sodium monensinate A (MonNa, **1**) with cobalt(II) or manganese(II) nitrate in acetonitrile/methanol solvent system results in the spontaneous formation of heteronuclear mixed-ligand coordination species [M(MonNa)_2_(NO_3_)_2_] (M = Co (**2**), Mn (**3**)). The structures **1**–**3** are determined by single-crystal X-ray diffraction. Furthermore, complexes **2** and **3** are investigated by infrared spectroscopy (IR), Time-of-Flight High-Resolution Mass Spectrometry (ToF HRMS), and elemental analysis (EA).

Single crystals from MonNa (**1**) were isolated as a side product during the crystallization procedure of [Hg(MonNa)_2_(SCN)_2_] earlier reported [37]. We discovered and analyzed ligand **1** by SCXRD weeks later after the above manuscript submission (Figure S1, CCDC 2389822). The structure of MonNa observed by us as acetonitrile clathrate is identical with that determined by Huczynski et al. (DEYGAQ [38]), which is already described in the literature. The data from DEYGAQ are collated with those obtained for complex **2** since the two SCXRD studies were performed at room temperature. On the other hand, as our crystals **1** originated from the same batch of antibiotic provided by the manufacturer Biovet Ltd. and used for the preparation of target complexes, we would like to share our findings at present and employ the resulting structural data for comparative purposes and the completeness of the research. An additional benefit is that our analysis of **1** is made at a low temperature (107 K) as the new structure **3** and bond lengths and angles can be paralleled at equivalent conditions.

### 2.1. Description of the Crystal Structures of Complexes ***2*** and ***3***

**Figure 2:**
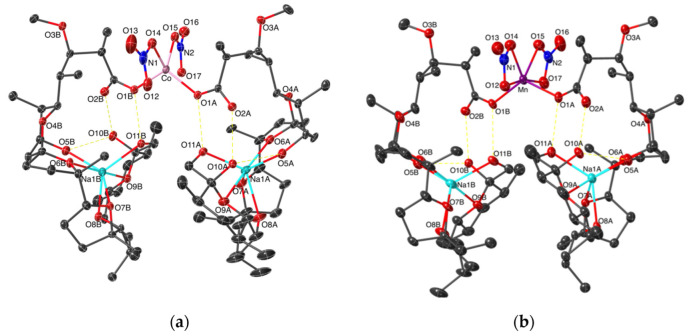
ORTEP drawings at 30% probability level of (**a**) Co(II) complex **2**, and (**b**) Mn(II) complex **3**. Protons are omitted for clarity. Color code: C—dark grey, O—red, N—blue, Na—cyan, Co—pink, Mn—purple, H-bonds—yellow.

ORTEP diagrams of [Co(MonNa)_2_(NO_3_)_2_] (**2**) and [Mn(MonNa)_2_(NO_3_)_2_] (**3**) are presented in Figure 2. The entire crystallographic data of compounds **2** and **3** are submitted to CCDC with numbers 2389824 and 2389823, respectively. The structures of the coordination species encompass a single divalent metal cation which interacts with sodium monensinates and nitrates to form electroneutral coordination species. Two of the primary places around Co(II) and Mn(II) centers are occupied by the unidentate carboxylate groups of MonNa with metal–oxygen bond lengths typical for such an environment (Table 1) [35,39,40,41]. The M-O2A and M-O2B contacts are of the order of 4 Å and exclude the bidentate coordination mode of the carboxylate anion. The monodentate fashion of COO-functions reflects the distances within this group with O1-C1 lengths in **2** and **3** being longer compared to the corresponding O2-C1 bonds (Table 2).

**Figure 3:**
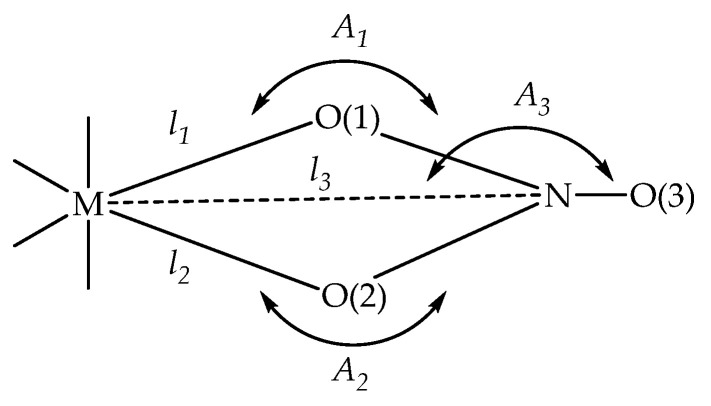
Depiction of nitrate group bound to the metal ion, where O(1) = O14, O15; O(2) = O12, O17; and O(3) = O13, O16 (the numbering of oxygen atoms follows that presented for complexes **2** and **3** in Figure 1), and *l*_1_ < *l*_2_ < *l*_3_.

**Figure 4:**
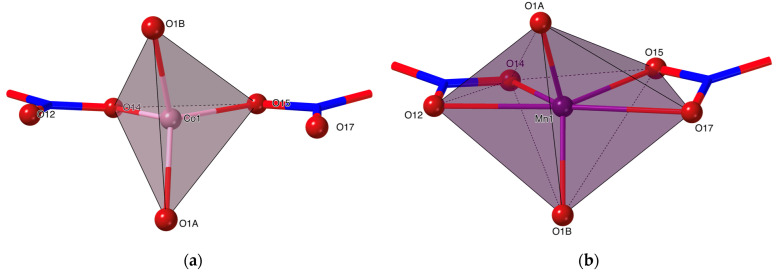
Internal coordination sphere of (**a**) Co(II) complex **2** and (**b**) Mn(II) complex **3**.

The arrangement of the two nitrate ligands differs in species **2** and **3**. For the sake of clarity, they will be further denoted as nitrates 1 (N1) and 2 (N2), bearing oxygens O12–14 and O15–17, respectively. In Co(II) complex **2**, the inorganic anions are definitely bound via O14 (N1) and O15 (N2), which are indistinguishable from each other and lay within the range characteristic of the Co-ONO_2_ set [42,43,44,45,46,47,48,49,50]. The significantly longer Co-O12 (N1) and Co-O17 (N2) contacts raise the question of whether these bonds can be treated as actually existing, i.e., whether the nitrate ligands are bound in a mono- or bidentate fashion to the cobalt(II) center. To solve this task, we applied the criteria introduced by Reedijk et al. [51] (Figure 3, Table 3) for distinguishing the coordination mode of the nitrate group in metal(II) complexes. The data analysis reveals that nitrate ligand 1 only interacts with Co(II) through its O14-atom. On the other hand, nitrate 2 could be defined as bound in a highly unsymmetrically bidentate (anisobidentate) mode via O15 and O17, with values of the Reedjik criteria being much closer to that of the monodentate fashion (Table 4). For that reason, we neglect the interactions Co-O12 and Co-O17 and describe the primary coordination core of cobalt(II) complex **2** as a CoO_4_ entity with a central metal ion placed in a slightly distorted tetrahedral environment (Figure 4a).

The inorganic nitrate 1 in Mn(II) complex **3** binds to the metal cation in a symmetrically bidentate mode (O14 and O12) with values of the bond distances characteristic of Mn-O_2_NO in the case of the chelating nitrato-group [52,53,54]. The higher value of the Mn-O17 bond distance in nitrate 2 compared to other Mn-O lengths prompted us to apply the Reedjik criteria again. As can be seen (Table 4), in this complex, the nitrate group 2 is involved in unsymmetrically bidentate coordination, whose parameters are very close to the symmetrical bidentate fashion. Based on such a comparison, we assume that both nitrates in complex **3** are bound bidentately, depicting the internal coordination sphere of the Mn(II) ion as a highly distorted octahedron bearing the MnO_6_ unit (Figure 4b).

As observed previously in our experiments on the coordination behavior of sodium monensinate [35,36,37], the hydrophilic cavity of the antibiotic is well-suited to accommodate sodium cations. They remain tightly bound to oxygen atoms when pre-hosted by monensinate A, and cannot be exchanged by metal(II) competitors under conditions used for obtaining species **2** and **3** (Table 2). The comparison between the structures of **2** and MonNa [38], solved at room temperature, and between **3** and **1** (at 107 K) reveals that the monovalent cation is irregularly six-fold coordinated within the monensinate cavity. Based on the convention, that two bond lengths can be distinguished if their difference is greater than three times the weighted standard deviation, it is apparent that the Na-O5H and Na-O6 bond distances do not change significantly upon the complexation of MonNa with Co(II) and Mn(II) ions. On the other hand, the Na-O7, Na-O8, and Na-O11H bonds are shorter and Na-O9 is longer compared to the parent structures with the antibiotic holding almost the same overall conformation in the corresponding subunits A and B of the complexes. In contrast to [M(MonNa)_2_Cl_2_] (M = Mn, Co, Cu) [35,36] and [Hg(MonNa)_2_(SCN)_2_] [37], where the Na-O distances are identical in both antibiotic subunits due to the different symmetry of the compounds, these in complexes **2** and **3** deviate slightly between A and B fragments, remaining in the same range typical of sodium–oxygen interactions. The accumulated structural data indicate that MonNa can be discussed as a single rigid building block which does not change drastically when it is complexed with heavy metal(II) cations.

The two ends of each sodium monensinate A in **2** and **3** are joined together by the intramolecular hydrogen bonds O1···HO11 and O2···HO10 (Table 5). The strong folding of the polyether chain is ensured by an additional interaction between HO10 and HO5 like in the crystal structures of MonNa. The coordination of O1 with divalent metal cations in species **2** and **3** leads to the significant lengthening of the O1···HO11 distances, while the others are kept in the same order as in the parent antibiotic. No intermolecular H-bonds are observed in the crystal packing of complexes **2** and **3**.

Having in hand the SCXRD data of the cobalt(II) and manganese(II) complexes of MonNa with nitrate and chloride co-ligands, we also performed powder XRD studies on the corresponding bulky samples (Figure S2). The experimental results disclose the potential of the powder technique: (i) it is a powerful tool for confirming the content and purity of the entire material isolated from the crude reaction mixture; (ii) it is useful in discriminating between the mixed-metal mixed-ligand coordination species of monensin containing NO_3_^−^ or Cl^−^ anions; and (iii) it can serve as a direct proof for the structural characterization of unknown isostructural coordination species with different metal(II) ions obtained under similar reaction conditions.

### 2.2. Spectral Characterization of Complexes **2** and **3**

The IR spectrum of sodium monensinate A (Figure S3) consists of a broad band in the range of 3550–3300 cm^−1^ assigned to the stretches of hydroxyl groups. The presence of a non-coordinated carboxylate anion engaged in intramolecular hydrogen bond formation is evident from the asymmetric (1562 cm^−1^) and symmetric (1409 cm^−1^) vibrations of its carbonyl group (Δ = 153 cm^−1^). In the IR spectra of complexes, the signals at ca. 3560, 3430, and 3255 cm^−1^ (ν_OH_) are characteristic of monensinate OH-functions participating in donor–acceptor interactions with sodium ion and/or intramolecular hydrogen bond formation. The absorbance at 3557 cm^−1^ (**2**) and at 3555 cm^−1^ (**3**) most probably can be assigned to the stretching of the primary hydroxyl groups at the tail of the ligand molecules. The participation of the deprotonated carboxylic moiety of monensin in the formation of **2** and **3** is evident by the corresponding COO-stretches visualized as an intense signal at 1605 cm^−1^ ($\nu_{COO}^{as}$) and a weak band at 1425 cm^−1^ ($\nu_{COO}^{sym}$). The difference of 180 cm^−1^ between the two vibrations is in agreement with the so-called “pseudo-bridging” arrangement of the carboxylate anion which is monodentately bound to the metal(II) center, and the non-coordinated oxygen is involved in intramolecular hydrogen bond formation [55,56,57,58]. The presence of nitrate ligands is apparent from the bands assigned to valence vibrations of non- and coordinated anions as follows: $\nu_{NO}$—1502/1493 cm^−1^ (**2**), 1493/1485 cm^−1^ (**3**), and $\nu_{ONO}^{as}$—1297/1274 cm^−1^ (**2** and **3**), respectively.

**Scheme 1:**
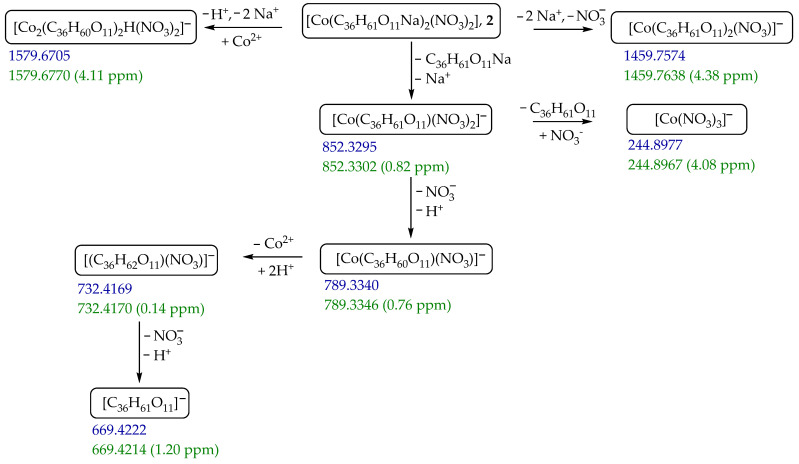
Fragmentation of complex **2** in a negative ESI mode. The blue numbers show the observed mass in *m*/*z* units and the green numbers show the calculated mass, followed by the error in parenthesis:

The ToF HRMS spectra of coordination species **2** and **3** in a positive Electro Spray Ionization (ESI+) mode dominate by peaks assigned to fragments containing sodium monensinate A ([MonNaH]^+^, [(MonNa)_2_H]^+^ for **2** (Figure S4) and **3**, and, in addition, [MonNa_2_]^+^ and [(MonNa)_2_Na]^+^ for **3**, Figure S5). No signals attributed to metal(II)-containing coordination species are observed. In contrast, the MS spectrum of complex **2** (in a negative mode, ESI−) is richer in peaks (Figure S6) arising from various cobalt(II)-bearing monensinate A fragments shown in Scheme 1, while that of species **3** consists of a single peak attributed to [Mon]^−^ (Figure S7).

$$error\;\left[\mathrm{ppm}\right]=\frac{\left|\left(m/z_{calc}-m/z_{exp}\right)\right|}{m/z_{calc}}\times 10^{6}.$$

### 2.3. Antimicrobial Properties of ***1**–**3***

The activity of sodium monensinate and its complexes **2**–**3** against Gram-positive microorganisms was evaluated by the agar diffusion assay. An additional effect to consider is the influence of the counterion which may change the biological properties of the coordination compounds. For that reason, the efficacy of the chloride complexes of MonNa with Co(II) and Mn(II) ions was also determined in the present study under the same experimental conditions. Among the target strains (Table 6), that of *B. cereus (BC)* appears to be the most sensitive to MonNa, and *K. rhizophila (KR)* is the most resistant. In between, the rest of the bacteria follow the order *S. aureus (SA*) = *S. saprophiticus (SS)* > *B. subtilis (BS)*. From the obtained data, no unequivocal conclusion can be drawn about the effect of the tested metal complexes on the growth of microorganisms. Generally, the coordination species are more efficient inhibitors than the parent ligand except the case of *BC*. The activity of modified monensinates is twice as high as MonNa towards *SA* and *SS* and is significantly better against *BS* and *KR*. The first effect could be explained by the inclusion of two moles of the active ligand per mole of the complex. On the other hand, the enhanced efficacy of the complexes in the latter case is obviously not a result of synergism between the constituent parts of the compounds, because the starting metal salts are ineffective, even when applied at high concentrations (MIC > 2 mM). The incorporation of chloride or nitrate anions appears to be indistinguishable and probably does not contribute significantly to the antimicrobial activity of the entire complexes. In order to answer the question of how the metal ions influence the antibacterial mode of action of the ligand, we need to accumulate more experimental data; therefore, a detailed targeted study involving a larger set of microorganisms is forthcoming.

## 3. Materials and Methods

### 3.1. Reagents and Materials

Sodium monensinate (MonNa, p.a.) was gifted by Biovet Ltd. (Peshtera, Bulgaria). The metal(II) nitrates ((Co(NO_3_)_2_ × 6H_2_O, and Mn(NO_3_)_2_ × 4H_2_O)), acetonitrile (MeCN), and methanol (MeOH) (p.a. grade) were delivered from local suppliers.

The Gram-positive bacteria and nutrient agar were purchased from the National Bank for Industrial Microorganisms and Cell Cultures (NBIMCC, Sofia, Bulgaria). The following non-pathogenic strains were studied at present: *Bacillus subtilis* (*BS*, NBIMCC 1050, ATCC 11774), *Bacillus cereus* (*BC*, NBIMCC 1085, ATCC 11778), *Kocuria rhizophila* (*KR*, NBIMCC 159, ATCC 9341), *Staphylococcus aureus* (*SA*, NBIMCC 509, ATCC 6538), and *Staphylococcus saprophyticus* (*SS*, NBIMCC 3348). Nutrient agar (pH 7.2–7.4) containing meat extract (3 g/L) and peptone (5 g/L) was used as culture media. The sterile plastic ware was supplied by local companies. Distilled water was used when required.

### 3.2. Synthesis of Complexes ***2*** and ***3***

Solution of metal(II) nitrate (0.2 mmol in 2 mL MeCN, Co(NO_3_)_2_ × 6H_2_O—58.2 mg, Mn(NO_3_)_2_ × 4H_2_O—50.2 mg) was added dropwise to a solution of MonNa (0.2 mmol in 3 mL MeCN/1 mL MeOH, 138.6 mg). The resulting mixtures were continuously stirred for 30 min and left to slowly evaporate, producing the corresponding complexes as violet (**2**) or colorless (**3**) crystals. The solids were filtered off, washed with MeCN, and dried in a desiccator.

Complex **2**, [Co(MonNa)_2_(NO_3_)_2_]: MW 1568.67 g/mol. Calc. H, 7.84; C, 55.13; N, 1.79; Na, 2.93; Co, 3.76%. Found H, 7.61; C, 53.85; N, 1.78; Na, 3.42; Co, 3.96%. Yield: 105.2 mg (67%).

Complex **3**, [Mn(MonNa)_2_(NO_3_)_2_]: MW 1564.67 g/mol. Calc. H, 7.86; C, 55.27; N, 1.79; Na, 2.94; Mn, 3.51%. Found H, 7.58; C, 53.63; N, 1.86; Na, 3.67; Mn, 4.10%. Yield: 91.5 mg (58%).

### 3.3. X-Ray Crystallography

Data collection and structure solution were conducted at the X-ray Crystallographic Facility, Department of Chemistry and Biochemistry, North Dakota State University (NDSU), Fargo ND, USA. The crystals were mounted on a Bruker APEX-II CCD diffractometer for data collection at 107 K (**1**, **3**) or 296 K (**2**) (due to difficulties with cooling device at that time). The preliminary set of cell constants was calculated from the reflections collected from four sets of 30 frames which produced the initial orientation matrices. Data collection was carried out using IµS Cu Kα radiation with a detector distance of 4.0 cm. The randomly oriented region of reciprocal space was surveyed to the extent of one sphere and a resolution of 0.84 Å. Nineteen ω-scan sections of frames and φ-scan sections with 1.8° width were collected to achieve the desired completeness of 99.8%. The intensity data were corrected for absorption and decay [59]. The final cell constants were calculated from the xyz-centroids of the strongest reflections from the actual data collection after integration [60].

The structures were solved and refined by the SHELX [61] set of programs with Olex 2 v.1.5 software package [62]. The SHELXT [63] program was used to provide most of the non-hydrogen atoms and the full-matrix least squares/difference Fourier cycles were performed to locate the remaining non-hydrogen atoms. All non-hydrogen atoms were refined with anisotropic displacement parameters. All hydrogen atoms were placed in ideal positions and refined as riding atoms with relative isotropic displacement parameters. The data collection parameters and refinement information for the single-crystal X-ray diffraction experiments are summarized in Table 7. Details on the geometrical data can be found in CCDC files 2389822-2389224. The images are generated using CrystalMaker Software Ltd., Oxford, UK (www.crystalmaker.com accessed on 10 October 2024).

### 3.4. Physical Measurements

IR spectra were recorded on a Nicolet 6700 FT-IR, Thermo Scientific (Madison, WI, USA) in KBr pellets. ToF HRMS measurements were carried out on a Waters SYNAPT G2-Si Q-ToF instrument (Waters Corporation, Milford, MA, USA). The samples were dissolved in methanol and directly injected for mass spectrometry analysis in positive or negative ionization mode. ESI conditions were as follows: capillary potential 3.0 kV (ESI+) and 2.3 kV (ESI−), sample cone potential 40 V, temperature source 90 °C, desolvation temperature 250 °C, and desolvation gas flow 350 L/h. The observed range was set from 50 to 2000 *m*/*z*.

The C, H, and N analysis was performed on a Thermo Fisher Flashsmart CHN/O (Thermo Fisher Scientific, Walthman, MA, USA) using conventional technique. The metal complexes **2** and **3** exhibit lower carbon values within 1.4–1.6% compared to the theoretically predicted data because of the formation of stable metal carbides. Metal content was determined by AAS on a Perkin Elmer AAnalyst 400 (Walthman, MA, USA) using standard stock solution (1000 µg/mL, Merck, Darmstadt, Germany). Working reference solutions were prepared after suitable dilution in appropriate solvent mixture.

### 3.5. Antibacterial Activity

The double-layer agar diffusion method was carried out on Petri dishes (90 mm) containing sterile (10 mL) and inoculated (10 mL, 1.5% inoculum, McFarland 4, A_650_ = 0.8–1) agar layers. After agar solidification, the holes of 6 mm/diameter were filled with 20 µL of the target compounds dissolved in MeOH (MonNa, complexes **2** and **3**, [Co(MonNa)_2_Cl_2_] and [Mn(MonNa)_2_Cl_2_] [35]). The tested solutions were obtained by subsequent two-fold dilutions of the stocks (1 mg/mL) up to 0.25 µg/mL. The diameter of the inhibited zones was measured 24 h after incubation at 30 °C. A total of nine readings from three separate experiments performed in triplicate were counted. Methanol served as a negative control. The minimum inhibitory concentration (MIC) of compounds causing the visible inhibition of the strain’s growth is calculated in µM units.

## 4. Conclusions

Two new heteronuclear mixed-ligand coordination species of the polyether ionophore sodium monensinate A with Co(II) (**2**) and Mn(II) (**3**) ions were synthesized and characterized. The observed stoichiometry of the analyzed compounds was proven to be equivalent to the composition [M(MonNa)_2_(NO_3_)_2_], which, in addition, is like already published structures of [M(MonNa)_2_Cl_2_] with these transition metal cations. The data reveal that, even with the same framework, the nitrate complexes extended the knowledge about the first coordination sphere of the central metal ions, due to the ability of the inorganic anion to serve as either a mono- (**2**) or a bidentate (**3**) ligand. Thus, the local symmetry differs significantly for the manganese and cobalt complexes from distorted octahedral to tetrahedral coordination, respectively. Here, we expanded our investigation with a powder diffraction analysis of bulky products of all complexes (including chloride compounds and the parent MonNa ligand). As was underlined in the results and discussion, this is the first important step for confirming the content and purity of the entire material isolated from the crude reaction mixture, before involving it in biological tests. The data from those assays, regarding the biological activity of target compounds against five bacterial strains, reveal that the impact of the counterion (chloride vs. nitrate) is similar and probably does not significantly influence the antimicrobial activity of the complexes. On the other hand, the stronger inhibitory effect of the coordination species containing Co(II) or Mn(II) ions on the growth of some microorganisms disclose their potential for the achievement of a better performance with lower concentrations applied, and the possibility to diminish the adverse effects of the sodium monensinate A application.

## Figures and Tables

**Table 1:** Important bond lengths (Å) and angles (°) in the inner coordination sphere of the metal(II) cation in species **2** and **3**.

	[Co(MonNa)_2_(NO_3_)_2_], 2	[Mn(MonNa)_2_(NO_3_)_2_], 3
M-O1A	1.985(5)	2.057(4)
M-O1B	1.978(5)	2.077(4)
M-O12	2.682(7)	2.377(5)
M-O13	3.903(6)	3.902 (6)
M-O14	1.997(5)	2.202(5)
M-O15	2.012(6)	2.149(5)
M-O16	3.876(6)	3.928(5)
M-O17	2.564(6)	2.489(5)
M-N1	2.736(7)	2.680(7)
M-N2	2.671(7)	2.725(6)
O1A-M-O1B	112.8(2)	121.5(2)
O1A-M-O14	114.9(2)	107.8(2)
O1A-M-O15	117.9(2)	104.0(2)
O1B-M-O14	109.7(2)	117.8(2)
O1B-M-O15	108.8(2)	117.6(2)
M-N1-O13	160.1(8)	172.6(6)
M-N2-O16	164.5(6)	169.7(6)
M-O12-N1	79.4(5)	89.0(4)
M-O14-N1	110.9(5)	97.5(4)
M-O15-N2	105.9(5)	102.2(4)
M-O17-N2	81.0(4)	86.8(4)

**Table 2:** Selected bond distances (Å) in parent MonNa and in MonNa ligands involved in the formation of **2** and **3**.

Bond	MonNa [38], 295 K	Complex 2, 296 K	MonNa 1, 107 K	Complex 3, 107 K
		Subunits A/B		Subunits A/B
Na-O5H	2.341(2)	2.330(6)/2.349(6)	2.332(2)	2.349(5)/2.327(6)
Na-O6	2.328(1)	2.309(6)/2.312(5)	2.313(2)	2.319(4)/2.307(6)
Na-O7	2.603(1)	2.453(6)/2.482(5)	2.582(2)	2.489(5)/2.470(1)
Na-O8	2.450(1)	2.394(7)/2.383(6)	2.436(2)	2.376(5)/2.402(6)
Na-O9	2.409(1)	2.439(6)/2.459(6)	2.389(2)	2.457(5)/2.423(6)
Na-O11H	2.383(1)	2.329(6)/2.340(6)	2.370(2)	2.329(5)/2.313(5)
O1-C1	1.245(2)	1.281(9)/1.297(9)	1.255(3)	1.286(8)/1.284(7)
O2-C1	1.261(2)	1.246(9)/1.255(9)	1.266(3)	1.228(9)/1.231(8)

**Table 3:** Criteria for discrimination between pure uni- and bidentate coordination fashion of nitrate ligand; the values for anisobidentate mode lay within the corresponding intermediate range [51].

Criteria	Unidentate	Bidentate
*l*_2_–*l*_1_, Å	>0.6	<0.3
*A*_1_–*A*_2_, °	>28	<14
*l*_3_–*l*_2_, Å	<0.1	>0.2
*A*_3_, °	<162	>168

**Table 4:** Selected bond lengths (*l*_1_*–l*_3_, Å) and angles (*A*_1_–*A*_3_, °) of complexes **2** and **3** used for determination of the binding mode of nitrate group.

Co(II) Complex 2				Mn(II) Complex 3			
Nitrate 1		Nitrate 2		Nitrate 1		Nitrate 2	
*l*_1_: Co-O14	1.977	*l*_1_: Co-O15	2.012	*l*_1_: Mn-O14	2.202	*l*_1_: Mn-O15	2.149
*l*_2_: Co-O12	2.682	*l*_2_: Co-O17	2.564	*l*_2_: Mn-O12	2.377	*l*_2_: Mn-O17	2.489
*l*_3_: Co-N1	2.736	*l*_3_: Co-N2	2.671	*l*_3_: Mn-N1	2.680	*l*_3_: Mn-N2	2.725
*A*_1_	110.9		105.9		97.5		102.2
*A*_2_	79.4		81.0		89.0		86.8
*l*_2_–*l*_1_, Å	0.705		0.552		0.175		0.340
*A*_1_–*A*_2_, °	31.5		24.9		8.5		15.4
*l*_3_–*l*_2_, Å	0.054		0.107		0.303		0.236
*A*_3_, °	160.1		164.5		172.6		169.7

**Table 5:** Intramolecular H-bonds (Å) in MonNa and complexes **2**–**3** (the oxygen–oxygen distances less than 3 Å are presented).

Bond	MonNa [38], 295 K	Complex 2, 296 K	MonNa 1, 107 K	Complex 3, 107 K
		Subunits A/B		Subunits A/B
O1···HO11	2.554	2.760/2.695	2.539	2.673/2.729
O2···HO10	2.684	2.642/2.624	2.677	2.627/2.624
HO10···HO5	2.880	2.891/2.841	2.903	2.811/2.844
N1S···HO5	2.980	−	2.925	−

**Table 6:** Antimicrobial effect of MonNa and its heteronuclear complexes [M(MonNa)_2_X_2_] on visible growth of bacterial strains (M = Co^2+^, Mn^2+^; and X = NO_3_^−^, Cl^−^).

Bacterial Strain	Minimum Inhibitory Concentration, µM				
	*BS*	*BC*	*KR*	*SA*	*SS*
MonNa, **1**	90.0	2.8	180	45.0	45.0
[Co(MonNa)_2_(NO_3_)_2_], **2**	19.7	2.5	39.3	19.7	9.8
[Mn(MonNa)_2_(NO_3_)_2_], **3**	10.0	5.0	40.0	20.0	20.0
[Co(MonNa)_2_Cl_2_]	20.6	5.2	41.2	20.6	20.6
[Mn(MonNa)_2_Cl_2_]	10.3	5.2	41.3	20.7	20.7

**Table 7:** Crystal data and structure refinement for compounds **1**–**3**.

Crystal Data	MonNa × MeCN, 1	Complex 2	Complex 3
Chemical formula	C_38_H_64_NNaO_11_	C_73_H_123.5_CoN_2.5_Na_2_O_28_	C_72_H_122_MnN_2_Na_2_O_28_
*M*_r_	733.89	1589.15	1564.63
Crystal system, space group	Orthorhombic, P 2_1_ 2_1_ 2_1_	Monoclinic, P2_1_	Monoclinic, P2_1_
Temperature (K)	107.00	296.15	106.99
*a* (Å)	12.7546(3)	12.2955(3)	12.2549(3)
*b* (Å)	16.3176(4)	23.9817(6)	23.6776(6)
*c* (Å)	18.4699(5)	13.5662(4)	13.6701(3)
*V* (Å^3^)	3844.04(17)	3997.63(18)	3963.82(16)
*Z*	4	2	2
Radiation type, λ [Å]	Cu *K*α, 1.54178		
µ (mm^−1^)	0.844	2.465	2.134
Crystal size (mm^3^)	0.25 × 0.19 × 0.17	0.201 × 0.142 × 0.131	0.192 × 0.174 × 0.085
**Data collection**			
Diffractometer	Bruker APEX-II CCD		
Absorption correction	Multi-scan		
*T*_min_, *T*_max_	0.610, 0.753	0.572, 0.753	0.559, 0.753
No. of measured, independent, and observed [*I* > 2σ(*I*)] reflections	21,811, 6775, 6530	53,699, 13,879, 13,172	45,614, 14,062, 11,472
*R*_int_	0.0475	0.0555	0.0720
**Refinement**			
*R*[*F*^2^ > 2σ(*F*^2^)], *wR*(*F*^2^), *S*	0.0346, 0.0895, 1.028	0.0694, 0.1781, 1.088	0.0604, 0.1416, 1.022
No. of reflections	6775	13879	14062
No. of parameters	478	1003	1071
No. of restraints	0	7	40
Δρ_max_, Δρ_min_ (e Å^−3^)	0.27/−0.34	0.45/−0.70	0.46/−0.47
	Flack x determined using quotients [(I+)-(I−)]/[(I+)+(I−)] [64]		
Abs. str. param.: Flack Hooft	−0.02(9) −0.03(4)	0.042(6) −0.0168(15)	0.020(6) 0.019(8)

## Data Availability

Data are available from the authors upon request.

## Supplementary Materials

The following supporting information can be downloaded at: https://www.mdpi.com/article/10.3390/ijms252212129/s1.
