# Supplementary material for: Cobalt(II) and Manganese(II) Complexes of Sodium Monensinate A Bearing Nitrate Co-Ligands
Source: Int J Mol Sci. 2024 Nov 12;25(22):12129. doi: 10.3390/ijms252212129 (PMC11593825; doi:10.3390/ijms252212129)
Supplement: Supplementary file 1 [file ijms-25-12129-s001.zip › ijms-3287176-supplementary.pdf]

Cobalt(II) and Manganese(II) Complexes of Sodium Monensinate A Bearing Nitrate Co-Ligands

Supplementary Information

Nikolay Petkov [1], Miroslav Boyadzhiev [1], Nikita Bozhilova [2], Petar Dorkov [3], Elzhana Encheva [1, 4], Angel Ugrinov [5*] and Ivayla N. Pantcheva [1*]

[1] Faculty of Chemistry and Pharmacy, Sofia University "St. Kliment Ohridski", 1164 Sofia, Bulgaria; ahnp@chem.uni-sofia.bg (N.P.), mirobo100@gmail.com (M.B.), eencheva@ipc.bas.bg (E.E.)
[2] Faculty of Biology, Sofia University "St. Kliment Ohridski", 1164 Sofia, Bulgaria; nikitobojilova@gmail.com
[3] Research and Development Department, Biovet Ltd., 4550 Peshtera, Bulgaria; p_dorkov@biovet.com
[4] Institute of Physical Chemistry, Bulgarian Academy of Sciences, 1113 Sofia, Bulgaria
[5] Department of Chemistry and Biochemistry, North Dakota State University, Fargo, ND 58105, USA
* Correspondence: angel.ugrinov@ndsu.edu (A.U.), ahip@chem.uni-sofia.bg (I.P.)

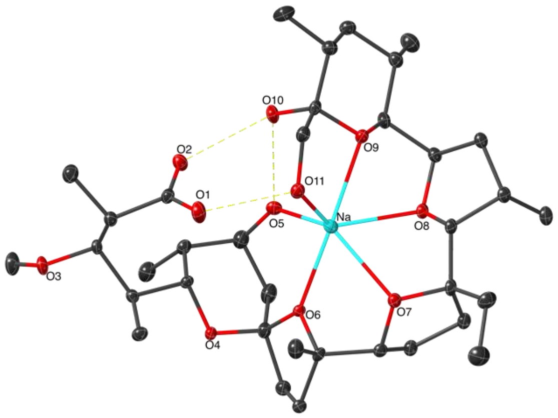

Figure S1. ORTEP drawing of MonNa (**1**) at 30% probability level with a numbering scheme. Protons and acetonitrile molecule are omitted for clarity. Colour code: C – dark grey, O – red, Na – cyan, H-bonds - yellow.

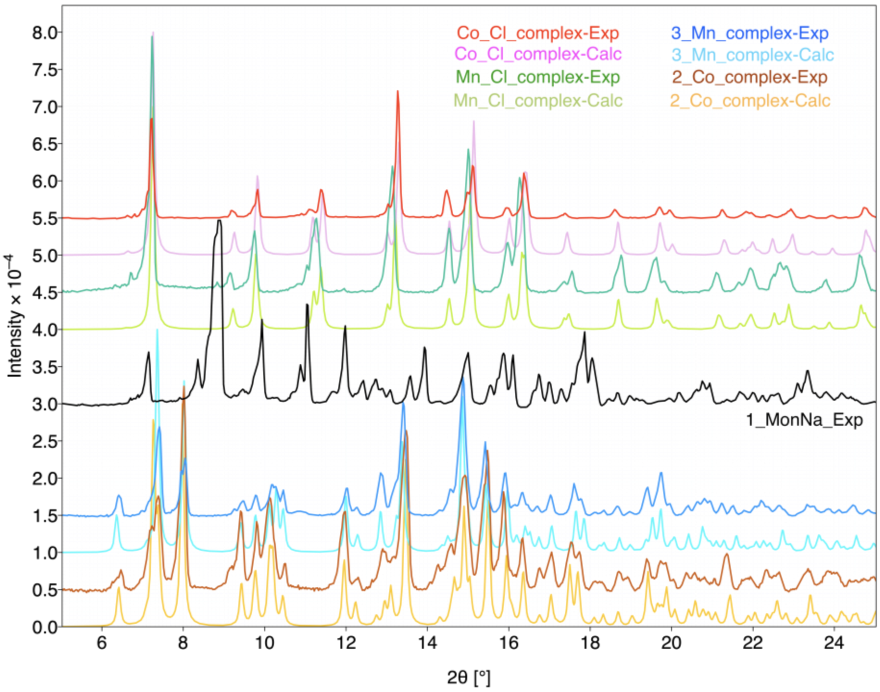

Figure S2. Experimental (Exp) and calculated (Calc) powder XRD spectra of MonNa and its Co(II)/Mn(II) complexes bearing chloride or nitrate anions as co-ligands. The following abbreviations were used: 1_MonNa – MonNa (**1**), 2_Co_complex – $[Co(MonNa)_2(NO_3)_2]$ (**2**), 3_Mn_complex – $[Mn(MonNa)_2(NO_3)_2]$ (**3**), Co_Cl_complex – $[Co(MonNa)_2Cl_2]$, Mn_Cl_complex – $[Mn(MonNa)_2Cl_2]$.

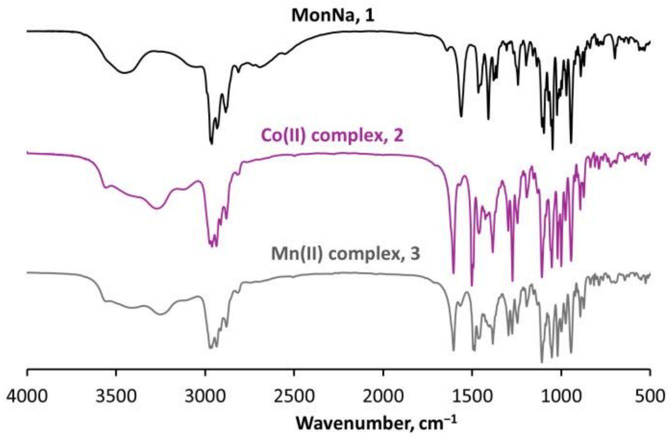

Figure S3. IR spectra of MonNa (**1**) and complexes **2**-**3** in KBr.

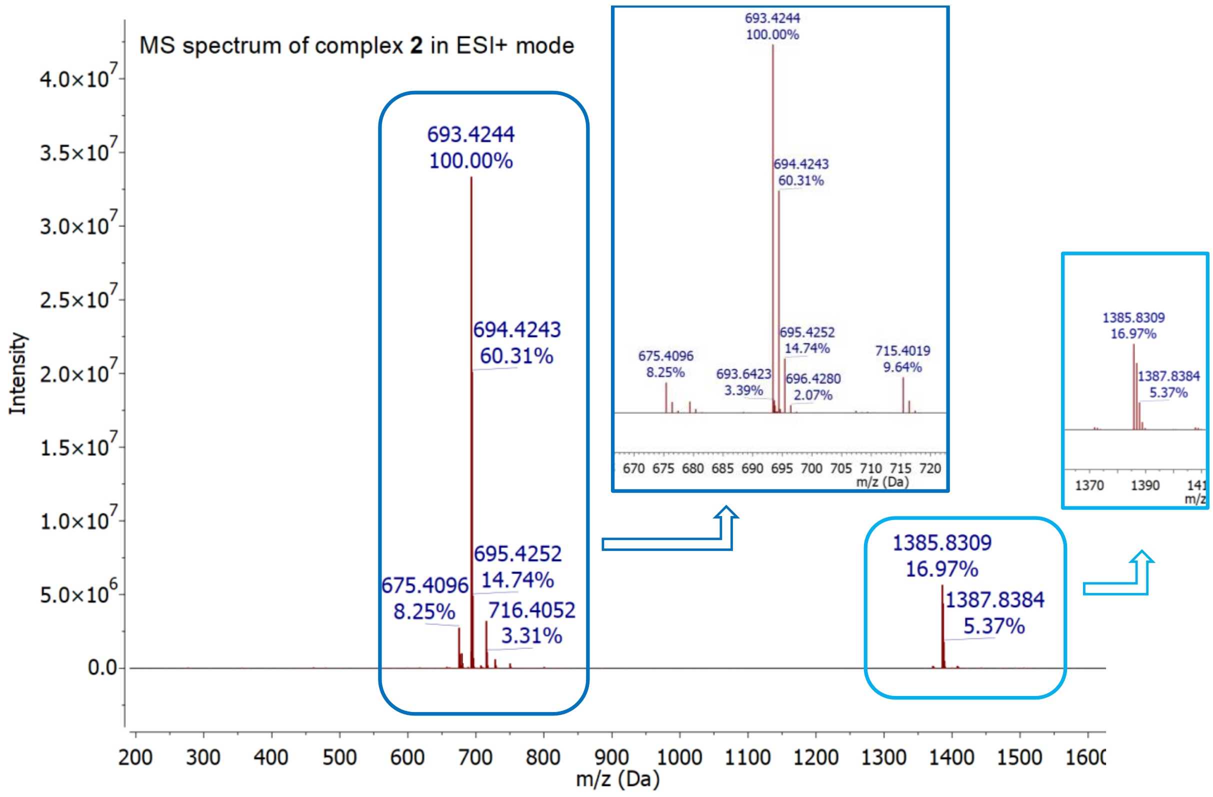

Figure S4. MS spectrum of complex **2** in ESI+ mode.
The main peaks are ascribed to the following ions:

ion	exp. m/z	calc. m/z	error [ppm]
[MonNa – H_2O + H]$^+$	675.4096	675.4084	1.78
[MonNa + H]$^+$	693.4244	693.4190	7.79
[MonNa + Na]$^+$	715.4019	715.4009	1.40
[$(MonNa)_2H$]$^+$	1385.8309	1385.8301	0.58

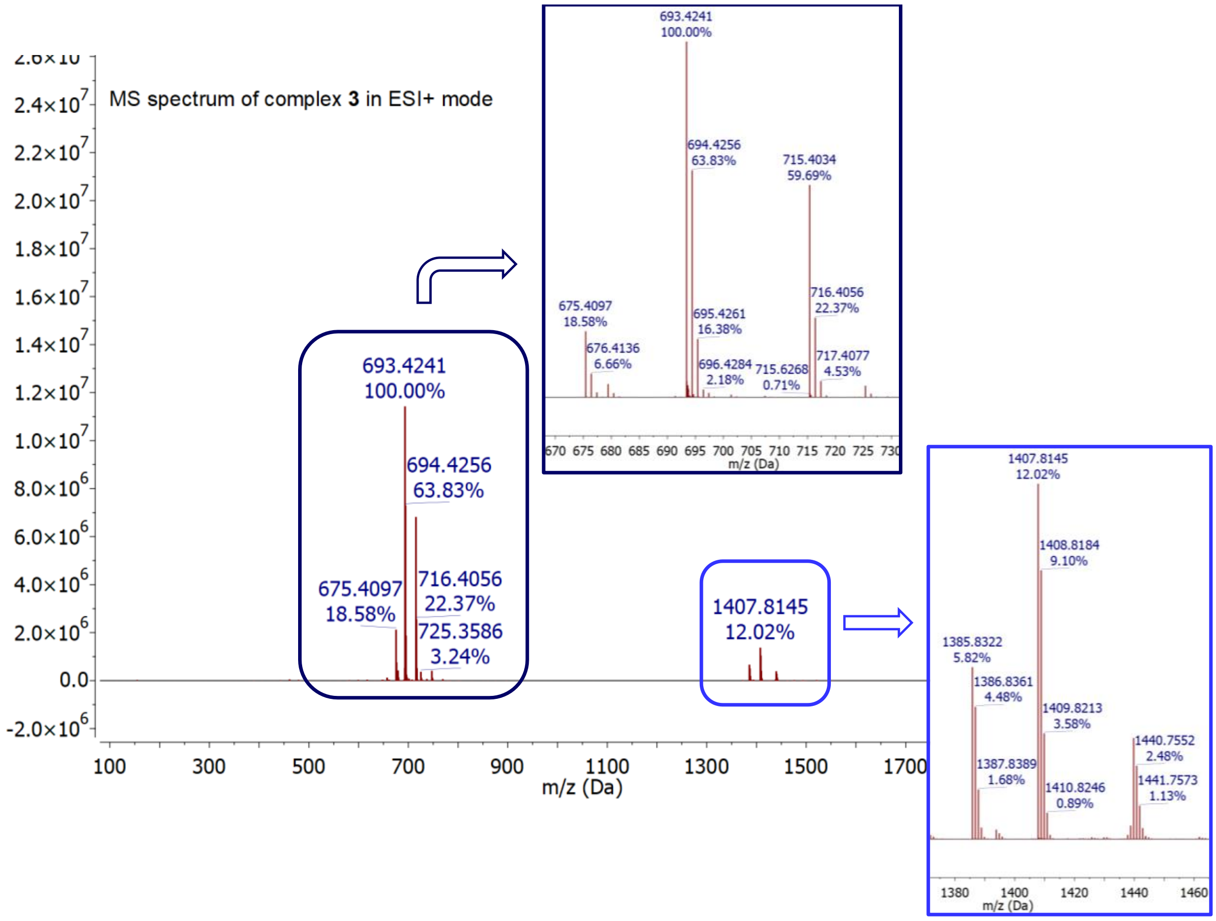

Figure S5. MS spectrum of complex **3** in ESI+ mode.

The main peaks are attributed to the following ions:

ion	exp. m/z	calc. m/z	error [ppm]
$[MonNa - H_2O + H]^+$	675.4097	675.4084	1.92
$[MonNa + H]^+$	693.4241	693.4190	7.35
$[MonNa + Na]^+$	715.4034	715.4009	3.49
$[(MonNa)_2H]^+$	1385.8322	1385.8301	1.52

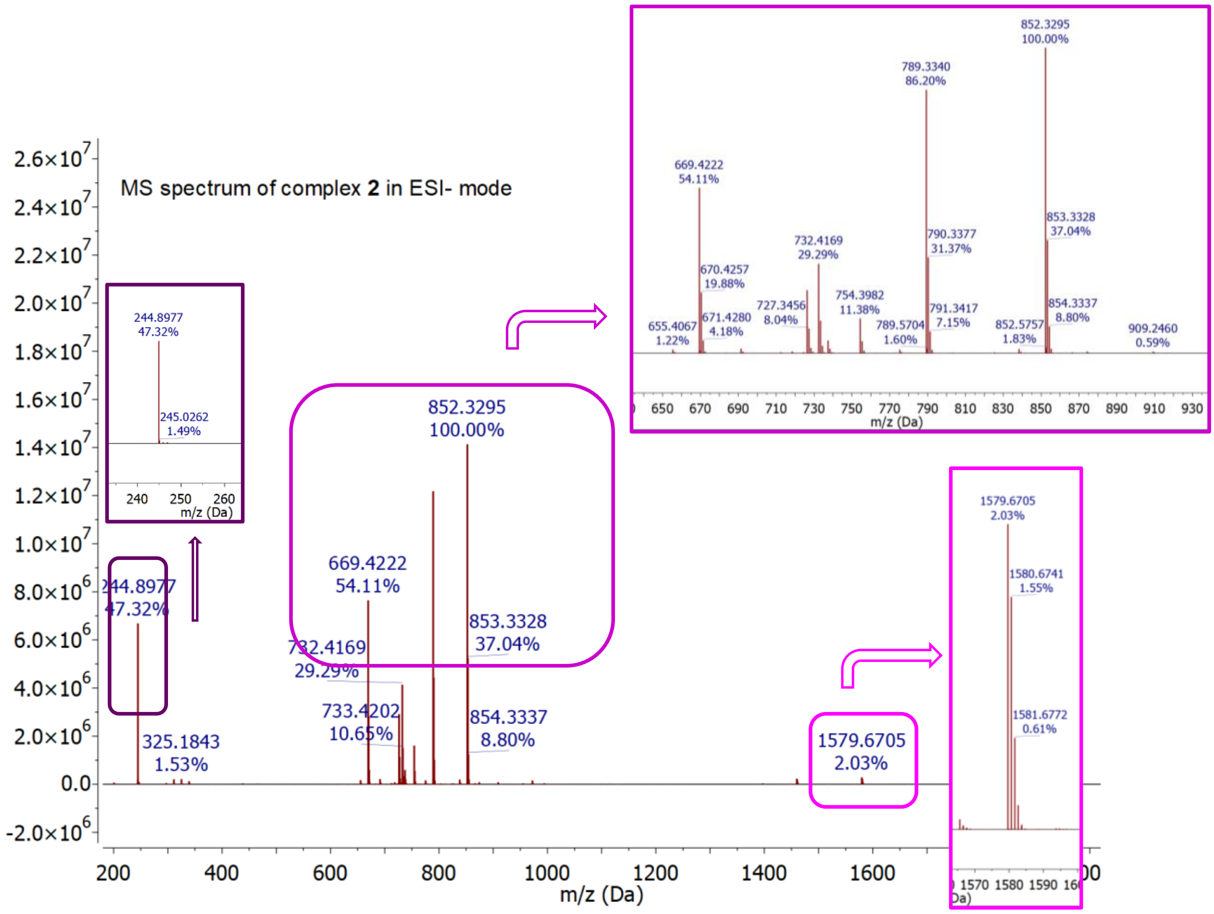

Figure S6. MS spectrum of complex **2** in ESI− mode (the assignment of the observed peaks is presented in Scheme 1 of the manuscript).

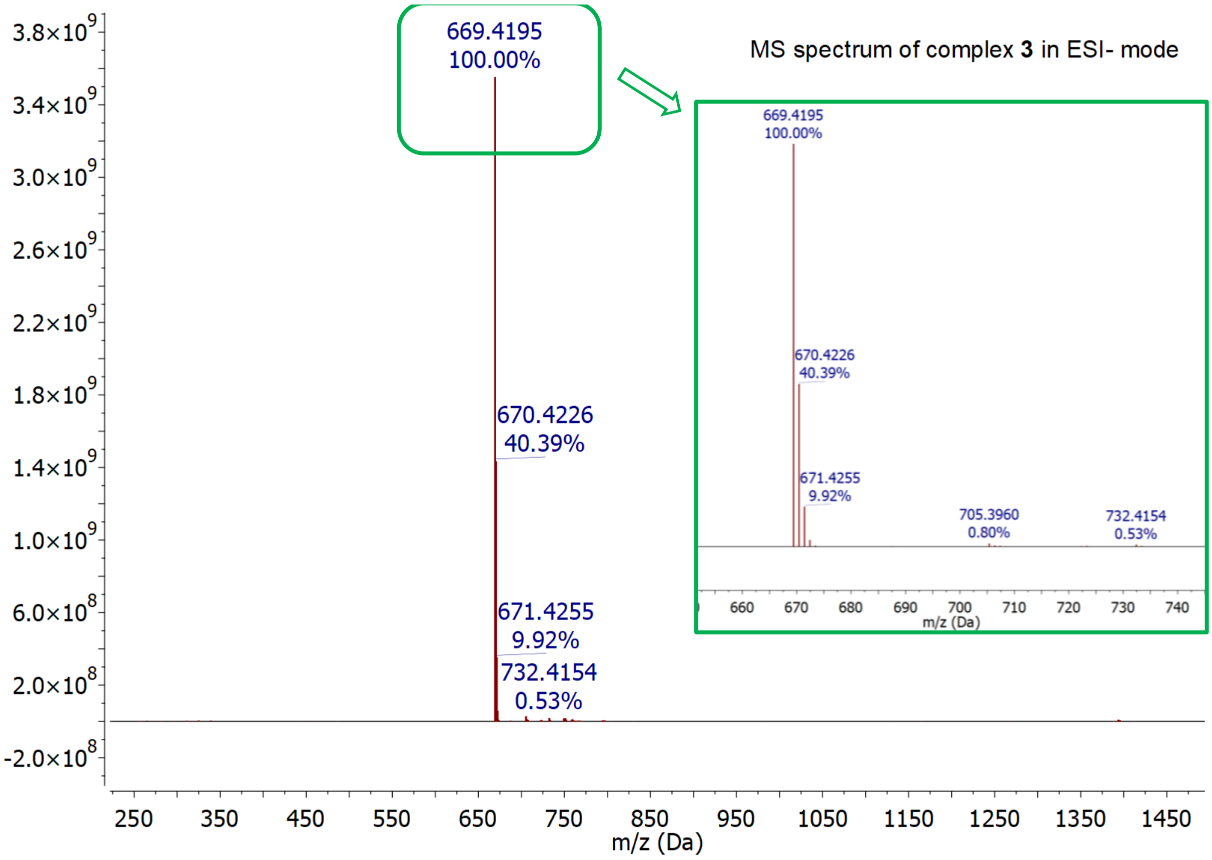

Figure S7. MS spectrum of complex **3** in ESI– mode (the peak at m/z 669.4195 belongs to [Mon⁻]; calc. m/z 669.4214, error 2.83 ppm).
